# Recombinant human diamine oxidase prevents hemodynamic effects of continuous histamine infusion in guinea pigs

**DOI:** 10.1007/s00011-023-01783-3

**Published:** 2023-10-09

**Authors:** Matthias Weiss-Tessbach, Birgit Reiter, Elisabeth Gludovacz, Thomas Boehm, Bernd Jilma, Marlene Rager-Resch

**Affiliations:** 1 Department of Clinical Pharmacology, Medical University Vienna, Waehringer Guertel 18-20, 1090 Vienna, Austria; 2 Department of Medicine I, Division of Infectious Diseases and Tropical Medicine, Medical University Vienna, Vienna, Austria; 3 Department of Laboratory Medicine, Medical University Vienna, Vienna, Austria; 4 Department of Biotechnology, University of Natural Resources and Life Sciences, Vienna, Austria

**Keywords:** (MeSH terms), Amine oxidase (Copper-containing), Diamines, Rodents, Heart rate, Blood pressure, Body temperature

## Abstract

**Objective:**

To test whether recombinant human diamine oxidase (rhDAO) with a mutated heparin-binding motif (mHBM), which shows an increased alpha-distribution half-life, prevents histamine-induced hemodynamic effects.

**Material:**

Thirty-eight female guinea pigs were either pretreated with rhDOA_mHBM or buffer.

**Treatment and methods:**

Guinea pigs received a continuous infusion of histamine. Heart rate (HR), body core temperature and mean arterial pressure (MAP) were measured and blood was collected.

**Results:**

Continuous intravenous infusion of 8 µg/kg/min histamine increased mean peak plasma histamine levels from 5 (± 0.3 SEM) to 28 ng/mL (± 4.9 SEM) after 30 min but had no effect on oxygen saturation. Guinea pigs pretreated with 4 mg/kg rhDAO_mHBM showed lower mean HR (*p* = 0.008), histamine plasma concentrations (*p* = 0.002), and higher body core temperatures at the end of the histamine challenge (*p* = 0.02) compared to controls. Cessation of histamine infusion led to a rebound increase in MAP, but this hemodynamic instability was prevented by rhDAO_mHBM. Pretreatment with 4 mg/kg rhDAO_mHBM reduced urinary histamine (*p* = 0.004) and 1-Methylhistamine (*p* < 0.0001) concentrations compared to controls.

**Conclusions:**

Prophylactic infusion of rhDAO_mHBM prevents hemodynamic effects in a guinea pig model of continuous histamine infusion. These findings might help in the translation from animals to humans and in the selection of the optimal dosing of rhDAO_mHBM during human histamine challenge studies.

**Supplementary Information:**

The online version contains supplementary material available at 10.1007/s00011-023-01783-3.

## Introduction

Histamine is one of the main mediators in anaphylaxis [1] and in acute infusion reactions to various drugs [2]. It is released from mast cells and basophils and leads to different severe systemic symptoms such as tachycardia, hypotension, and bronchoconstriction, but also flush or urticaria [3–5].

Currently, the state-of-the-art anaphylaxis treatment consists of fluid administration, vasopressors, supplemental oxygen, histamine 1 receptor (H1R) antagonists, and glucocorticoids [6, 7].

Adrenaline is recommended in severe reactions, although there is no evidence from randomized trials for its efficacy in anaphylaxis [1]. Intramuscular injection of epinephrine is used outside of a critical care setting to minimize potential fatal adverse effects associated with intravenous injections like pulmonary edema and cardiac complications [8, 9]. Nevertheless, myocardial infarctions were also observed after recommended doses of intramuscular epinephrine were administered to young patients in the absence of abnormal coronary arteries [10].

H1R antagonists are commonly used to treat anaphylaxis despite being insufficient and overwhelmed by the massive histamine release during anaphylactic reactions [11]. While the beneficial effects of H1R antagonists against allergic urticaria and rhinoconjunctivitis are undisputed, their positive influence on histamine-mediated anaphylactic shock and bronchoconstriction has not been demonstrated [12].

Glucocorticoids are administered to possibly prevent late-phase reactions. Their use in an acute setting is controversial because there is no evidence from high-quality studies to show benefits during acute anaphylaxis [7].

Therefore, new therapeutic approaches to counteract the underlying mediators of acute anaphylactic reactions are urgently needed. Histamine is an excellent target considering its central role in acute anaphylactic reactions. For instance, insect sting challenges inducing anaphylactic shock increased histamine plasma levels > 100-fold within minutes, and the histamine concentrations were inversely correlated to the drop in MAP [13]. Diamine oxidase is a copper-containing amine oxidase that rapidly degrades histamine and therefore represents a promising therapeutic approach for the treatment of severe anaphylactic reactions [14]. Treatment with rhDAO_mHBM reduced the histamine-induced decrease in body temperature and plasma histamine concentrations and prevented the development of severe clinical symptoms in mice [15]*.* However, most rodents including mice and rats are insensitive to histamine, which is demonstrated by the difference in the half-lethal dose (LD_50_) of intravenously administered histamine of 630 and 595 mg/kg for rats and mice, respectively, compared to 2 mg/kg for rabbits and likely much lower for humans [16, 17].

Guinea pigs are histamine sensitive with an LD_50_ of intravenous histamine of only 0.18 mg/kg [18]. The anatomy and physiology of guinea pig lungs are similar to human lungs. The respiratory tract of both species exhibits a hypersensitivity response to allergens mediated by histamine and leukotrienes via mast cells. Both humans and guinea pigs are prone to bronchoconstriction, unlike mice and rats, where histamine has no effect on smooth muscles of the respiratory tract [19].

The aim of this study is to establish a model of histamine-induced hemodynamic changes in guinea pigs to test whether rhDAO_mHBM can prevent cardiovascular responses. We hypothesized that rhDAO_mHBM would be able to rapidly inactivate toxic histamine concentrations in vivo.

## Material and methods

All experiments and procedures were performed within the Division of Biomedical Research, Medical University of Vienna, according to the animal protocol GZ 2021–0.209.778, approved by the Austrian Ministry of Education, Science, and Research.

### Animals, housing, and acclimatization

Thirty-eight female Dunkin Hartley guinea pigs (Charles River Laboratories, Sulzfeld, Germany) weighing 465–625 g were housed for at least 14 days prior to the experiment in groups of four in cages containing wooden bedding material. The guinea pigs were fed a commercially available pelleted diet (ssniff Spezialdiäten, Soest, Germany) ad libitum and hay daily. Tap water was available ad libitum. Once a week they received vitamin C (Gatt Koller GmbH, Absam, Austria) through drinking water. The animals were kept in a light/dark cycle of 12:12 h at 20 ± 2 °C and 55 ± 2% humidity.

### Surgical procedure

The guinea pigs were anesthetized subcutaneously (0.1 mg/kg medetomidine [Domitor, Provet, Switzerland], 1 mg/kg midazolam [Midazolam Accord, Accord Healthcare, Austria], 0.03 mg/kg fentanyl [Fentanyl Primal, Primal Critical Care, Netherlands] and 10 mg/kg ketamine [Ketanest, Pfizer, Austria]) by the animal keeper, who received the mixture from the veterinarian. The animals were returned to the cage until the righting reflex disappeared and then transported to the operating room. In preparation for surgery, the ventral side of the neck was shaved and disinfected, and a local anesthetic (lidocaine 1%, 4 mg/kg; Xylanest purum, Gebro Pharma, Austria) was injected subcutaneously. Animals were placed on a heating pad (37 °C) to prevent hypothermia and a rectal probe was inserted to monitor body temperature. Guinea pigs received 0.7 L/min of oxygen through a face mask and a pulse oximeter was attached to the hind limb to measure blood oxygen saturation. After the disappearance of the foot withdrawal reflex, a double-lumen catheter (Nutriline Twinflow 2Fr, Vygon, Germany) was inserted into the right or left jugular vein for intravenous administration of anesthesia and experimental compounds. To maintain anesthesia, guinea pigs were administered a mixture of etomidate (0.2 mg/kg/min; Etomidate-Lipuro, 2 mg/ml, Braun, Germany) and fentanyl (0.03 mg/kg/h; diluted to 20 µg/mL) via the jugular vein catheter as a continuous drip infusion. An additional catheter (Nabelkatheter 2.5 Fr, Vygon, Germany) was placed in the right or left carotid artery for invasive blood and HR measurements and for blood sampling (Supplementary Fig. 1). HR, MAP, and body core temperature (BT) were measured every minute or every three minutes and are presented after normalization to baseline.

Citrate blood (0.3 mL per timepoint, 3.8% sodium citrate) was collected before intravenous injection of test substances (minute 0) and thereafter every 10 min. For histamine measurements, an additional citrate blood sample (0.2 mL per timepoint, 3.8% sodium citrate) was collected every 10 min with the highly potent and specific DAO-inhibitor diminazene aceturate (D7770, Sigma-Aldrich, Austria) at a final concentration of 10 µM to prevent histamine degradation. After each blood collection, the arterial catheter was flushed with 0.3 mL alteplase (400 ng/mL diluted in NaCl, Actilyse Cathflo, Boehringer Ingelheim, Austria) to prevent clotting in the catheter lumen.

Arterial blood gases, acid–base status, electrolyte levels, oxygen status, and lactate were measured at minute 0 and at the end of each experiment using a blood gas analyzer (ABL800 Flex, Drott, Austria).

### Experimental procedure

#### Unfractionated heparin

After the first blood draw four guinea pigs received an intravenous bolus infusion of unfractionated heparin (500 IE/kg, Gilvasan Pharma, Austria) and blood was sampled after five minutes.

#### rhDAO_mHBM pharmacokinetics (PK)

Three guinea pigs received rhDAO_mHBM at a concentration of 2 mg/kg and were observed for 90–100 min. The generation and purification of rhDAO_mHBM were recently published [14].

#### Histamine dose finding

Four guinea pigs received a continuous drip infusion of histamine (#53300, Sigma-Aldrich, Austria). Histamine dosages ranged from 0.2 to 8 µg/kg/min and increased every 10 or 20 min until a measurable clinical response was observed. The observation period was 70–90 min.

#### DAO prophylaxis

Twelve guinea pigs received either rhDAO_mHBM (2 or 4 mg/kg in 0.05–0.1 mL/100 g of 24 mM HEPES and 73 mM potassium chloride) or buffer (0.05–0.1 mL/100 g of 24 mM HEPES and 73 mM potassium chloride) prophylactically into the artery after the first blood draw (minute 0). After a stabilization time of 10 min, a continuous drip infusion of 8 µg/kg/min histamine was administered for 30 min. At minute 40, the histamine infusion was stopped and the animals were observed until minute 60.

### DAO activity measurement

Guinea pig DAO activity from citrate plasma was measured as described [20]*.* Briefly, citrate plasma was diluted 1:10 in PBS and incubated for 120 min with 1 mM *ortho*-aminobenzaldehyde (A9628, Sigma-Aldrich, Austria) and either 200 µM cadaverine (#D22606 Sigma-Aldrich, Austria) or PBS. DAO-deaminated cadaverine autocyclizes to delta-1-piperideine and fuses with *ortho*-aminobenzaldehyde forming a fluorophore, which can be measured at EX440 and EM620 nm. DAO activity was converted into concentration units using an rhDAO standard curve ranging from 0 µg/mL to 5 µg/mL in baseline guinea pig plasma.

### Recombinant human DAO ELISA measurements in plasma

The concentrations of rhDAO_mHBM in the plasma of guinea pigs were measured using an ELISA as previously described [21]*.* Briefly, citrate plasma was diluted 1:80 in 1% human serum albumin in PBS and again 1:5 in low-cross buffer (#100050, Candor Bioscience, Germany). A standard curve using rhDAO_mHBM in baseline guinea pig citrate plasma was used for quantification. A monoclonal mouse anti-human-DAO antibody (a generous gift from Prof. Andrea Quaroni, Cornell University, Ithaca, NY, USA) was coated at 5 µg/mL in 50 mM carbonate buffer (pH 9.6) overnight and the microtiter plates were subsequently blocked using 1% bovine serum albumin in PBS. After washing with 0.1% Tween-20 in PBS (pH 7.4), the samples and standards were incubated at room temperature. A polyclonal rabbit anti-human-DAO serum IgG fraction (in-house generated; not commercially available) and an HRP-labelled donkey anti-rabbit antibody (#SAB3700928, Sigma-Aldrich, Austria) were used for detection. Additionally, 10 µg/mL donkey IgG was used to minimize the background signal (#017–000-003, Jackson ImmunoResearch, United Kingdom). The chemiluminescent substrate SuperSignal™ was used for signal generation (#37069, Thermo Fisher, Austria).

### Histamine measurements in the dose-finding phase

During the dose-finding phase histamine concentrations in citrate plasma containing 10 µM diminazene-aceturate were analyzed using the histamine homogeneous time-resolved fluorescence (HTRF) dynamic kit (62HTMDPET, PerkinElmer formerly Cisbio, France) according to the manufacturer’s instructions with a histamine standard curve in baseline guinea pig plasma.

Citrate plasma with 10 µM diminazene aceturate from all other guinea pigs was analyzed using the state-of-the-art enzyme immunoassay (EIA) for histamine quantification. The kit was used according to the instructions provided by the manufacturer (IM2562, Immunotech/Beckman Coulter, Czech Republic) with a histamine standard curve in guinea pig plasma. All measurements were performed in duplicate and all histamine concentrations refer to the histamine base.

### Histamine and 1-Methylhistamine measurements by liquid chromatography – tandem mass spectrometry (LC–MS/MS) in plasma and urine

A total of 38 guinea pigs were used for the establishment of surgical methods, substance titration (dose-finding phase), and the main experiment. Determination of analytes in the plasma and urine of guinea pigs was performed by staff members blinded to the treatment allocation. Histamine and 1-Methylhistamine were determined in all baseline urine samples, whereas plasma histamine was analyzed in 12 guinea pigs with seven time points included in the main experiment.

All samples were analyzed using LC–MS/MS in positive mode by multiple reaction monitoring (MRM). More detailed information on the analytical method can be found in the supplementary material.

### Statistical analysis

Statistical analyses were performed using GraphPad Prism Version 8.4.0. (GraphPad Software Inc., San Diego, CA, USA). Differences in DAO plasma concentrations were compared using a paired *t*-test. The area under the curves (AUCs) of individual HR, MAP, and plasma histamine concentrations during the course of an experiment were compared using a two-sided unpaired *t*-test without Welch’s correction. The increase in urinary histamine from minute 0 to 60 in guinea pigs receiving 8 µg/kg/min histamine was calculated using a paired *t*-test. Differences in urinary histamine excretion between guinea pigs receiving histamine alone and guinea pigs pretreated with 4 mg/kg rhDAO_mHBM were calculated using a two-sided unpaired *t*-test without Welch’s correction. Continuous data are presented as mean ± SEM. Statistical significance was defined as *p* < 0.05.

## Results

### Pharmacokinetics of recombinant human DAO with a mutated heparin-binding motif and heparin-induced release of endogenous DAO in guinea pigs

**Fig. 1:**
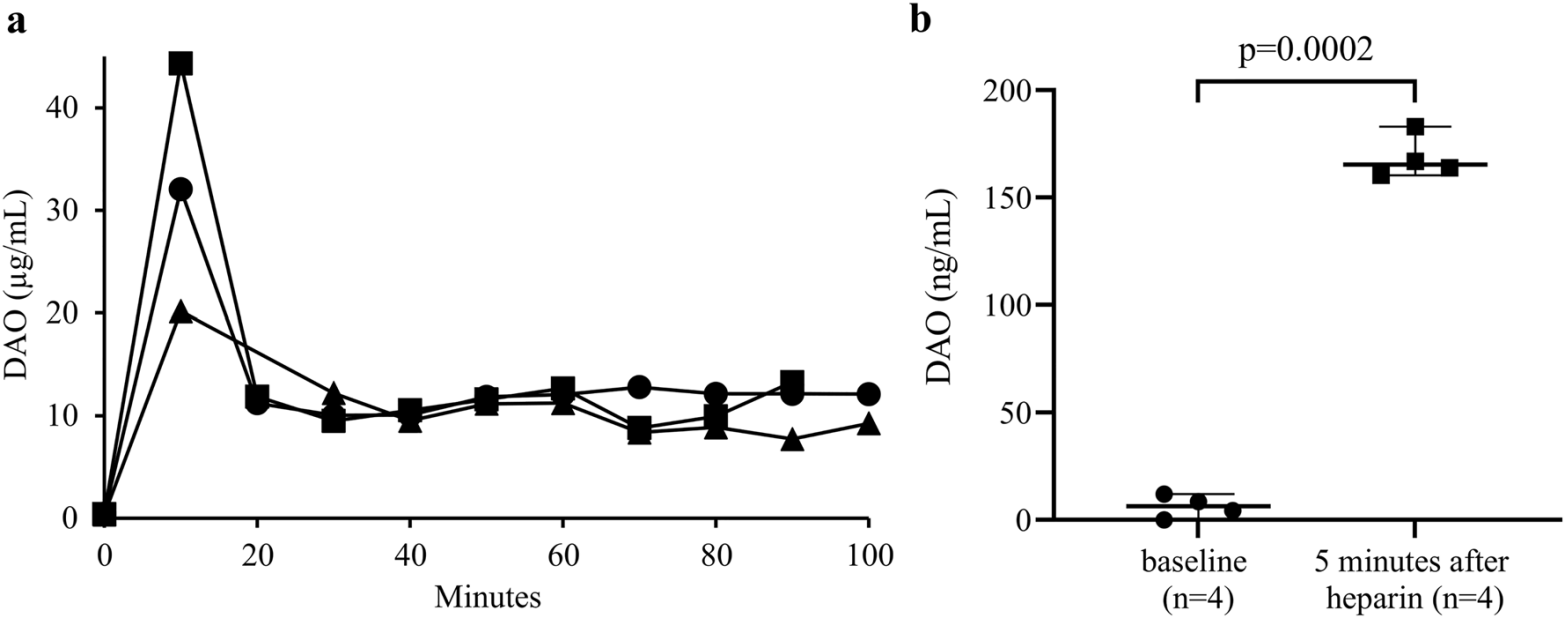
Concentrations of human diamine oxidase (DAO) after intravenous injection compared to endogenous DAO released after heparin infusion in guinea pigs. **a** Recombinant human (rh)DAO (2 mg/kg) with a mutated heparin-binding motif (rhDAO_mHBM) was infused into guinea pigs (*n* = 3) and blood was drawn every ten minutes for up to 100 min. Antigen concentrations of rhDAO_mHBM were determined using an ELISA. **b** DAO concentrations in guinea pig plasma (*n* = 4) before and five minutes after intravenous infusion of 500 IE/kg heparin were determined using a DAO activity assay with a standard curve spiking rhDAO into guinea pig plasma. Individual values and the mean of duplicates (± SEM) are shown. The heparin-induced DAO activity change was tested for significance using a two-sided paired *t*-test

Intravenous infusion of rhDAO_mHBM resulted in an initial peak, followed by a stable DAO antigen concentration between 7.7 and 13.3 µg/mL from 20 to 100 min (Fig. 1a). For comparison, the release of endogenous DAO was measured in the circulation of guinea pigs after intravenous injection of 500 IE/kg unfractionated heparin. Using a specific DAO activity assay, the concentration of enzymatically active guinea pig DAO increased 24 times from a mean baseline of 7 ng/mL to 170 ng/mL five minutes after heparin injection (*p* = 0.0002) (Fig. 1b).

### Dose-finding: Histamine dose-dependently increases the HR

**Fig. 2:**
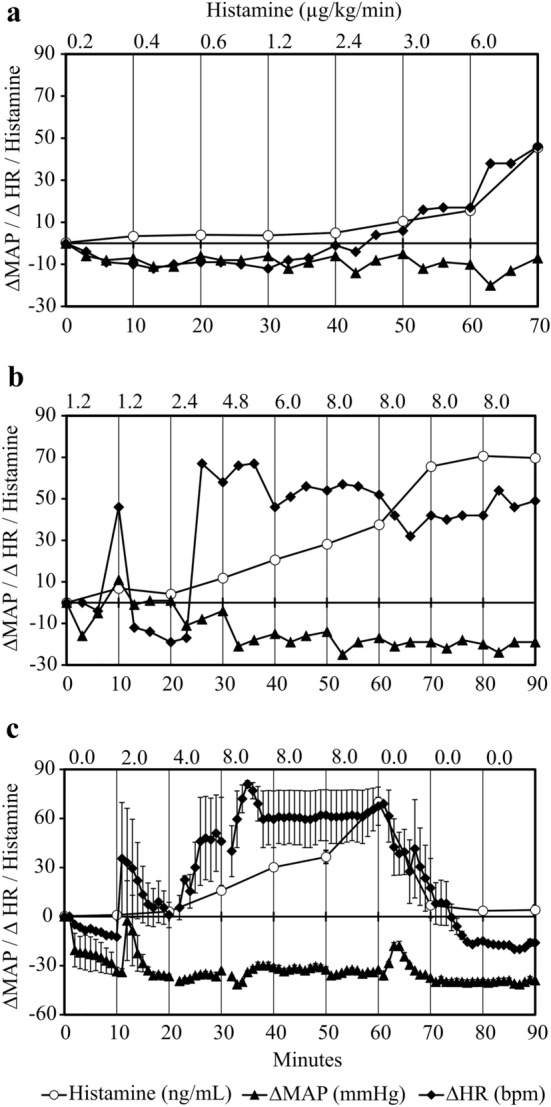
Histamine causes a dose-dependent reversibly increased HR. **a** One guinea pig received increasing infusion rates of histamine from 0.2 to 6 µg/kg/min (upper x-axis) with increments every 10 min (lower x-axis). **b** One guinea pig received an increasing amount of histamine starting with 1.2 up to 8 µg/kg/min as indicated by the upper *x*-axis. **c** Two guinea pigs received increasing amounts of histamine from 2 to 8 µg/kg/min (upper *x*-axis) for 50 min (lower x-axis). The histamine infusion was then stopped and the guinea pigs were observed for further 30 min. Data in Fig. 2c are presented as means of two guinea pigs. Plasma histamine concentrations were measured in duplicate and are shown as mean (± SEM). Changes in the heart rates (∆HR) and mean arterial pressures (∆MAP) are normalized to baseline and are shown as means (± SEM) on the *y*-axes

To find the histamine infusion rate necessary to cause a sustained clinical reaction in either the HR or the MAP, we started the continuous histamine infusion rate at 0.2 µg/kg/min. In the first guinea pig, the HR started to increase at 3 µg/kg/min and increased further at 6 µg/kg/min (Fig. 2a). In the second guinea pig, the HR increased sharply at 2.4 µg/kg/min and was sustained at a high level (> 30∆HR) after increasing the dose to 8 µg/kg/min (Fig. 2b). The third dose-finding setup was performed in two guinea pigs, which showed HR increments at 2 µg/kg/min, dropping after 10 min of continuous infusion but increasing again when infusing 4 µg/kg/min (20 min) and 8 µg/kg/min (30 min) (Fig. 2c). This effect of 4 and 8 µg/kg/min histamine on HR was rapidly reversible within 10 min when the histamine infusion was stopped. Consistent with this observation, histamine levels normalized within 10 min after histamine infusion was discontinued (Fig. 2c). The MAP decreased during the course of the experiments, but this effect did not appear to be reversible after stopping the histamine infusion (Fig. 2). The constant decrease in MAP was seen despite the fact that the amount of infused volume exceeded the blood loss by blood sampling by 30%.

### Recombinant human DAO protects guinea pigs from histamine-induced hemodynamic effects

**Fig. 3:**
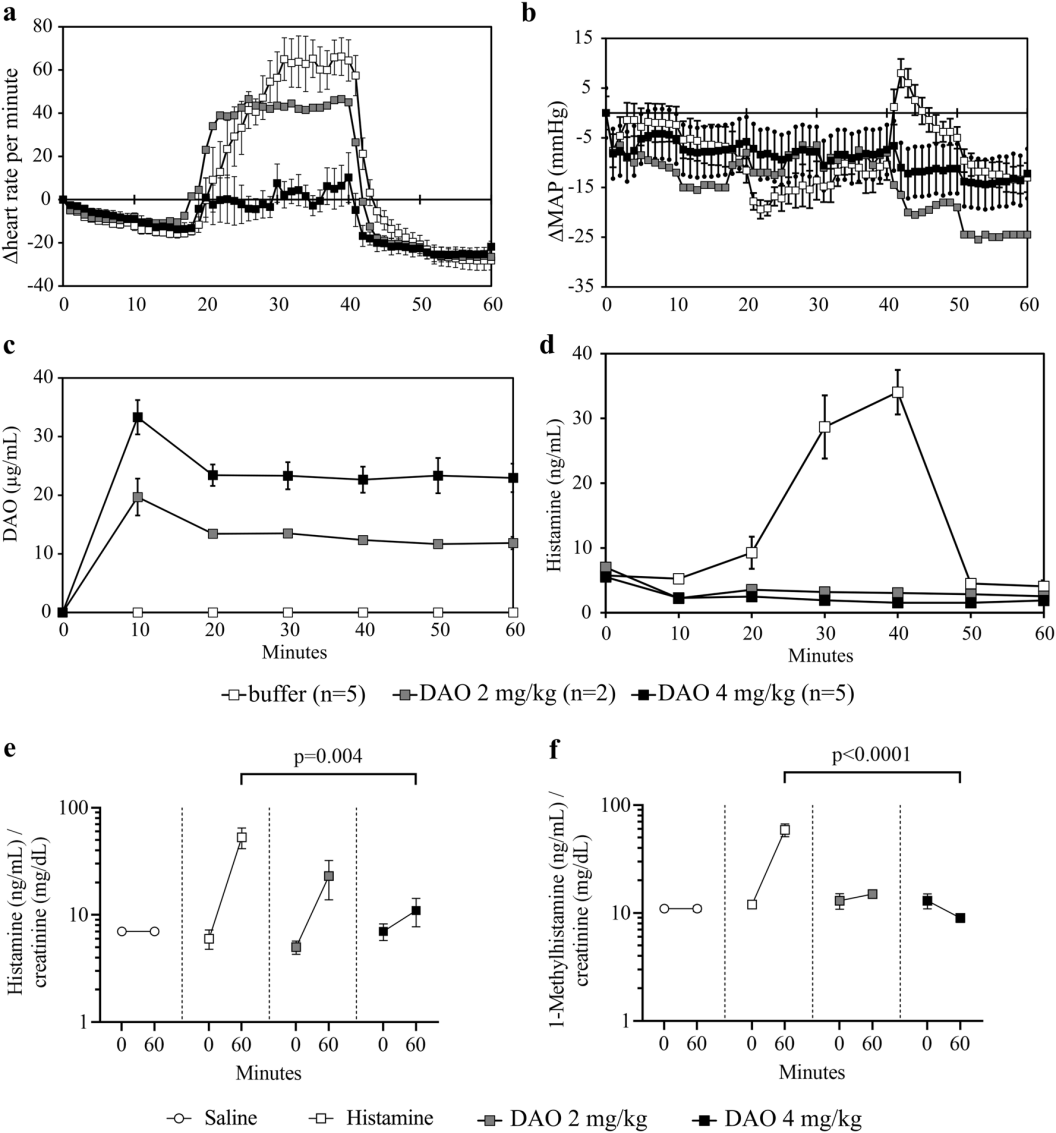
Recombinant human diamine oxidase (rhDAO) with a mutated heparin-binding motif (mHBM) protects guinea pigs from histamine-induced hemodynamic effects. Guinea pigs were pretreated with either 2 mg/kg (*n* = 2) or 4 mg/kg (*n* = 5) rhDAO with a mutated heparin-binding motif (rhDAO_mHBM) or buffer (*n* = 5) at minute 0. Histamine (8 µg/kg/min) was continuously infused from 10 to 40 min. **a** Differences in heart rate (∆HR, panel a) and mean arterial pressure (∆MAP, panel b) normalized to baseline are shown as mean (± SEM). **c** Plasma concentrations of rhDAO_mHBM were determined using ELISA in duplicate and are shown as mean ± SEM. **d** Plasma histamine concentrations were measured in duplicate and are shown as mean ± SEM. **e** rhDAO_mHBM dose-dependently decreased urinary histamine excretion. Urinary histamine concentrations were normalized to creatinine in guinea pigs receiving either saline (*n* = 38), histamine (8 µg/kg/min) (*n* = 6 at min 0, *n* = 5 at min 60), histamine (8 µg/kg/min) with 2 mg/kg rhDAO_mHBM (*n* = 2), or histamine (8 µg/kg/min) with 4 mg/kg rhDAO_mHBM (*n* = 6). Data are presented as means ± SEM. The difference in urinary histamine and 1-Methylhistamine excretion of guinea pigs receiving histamine alone and 4 mg/kg rhDAO_mHBM at minute 0 and minute 60 was calculated using an unpaired *t*-test without Welch’s correction

Pretreatment with rhDAO_mHBM using 2 mg/kg (mean baseline HR: 212/min) did not sufficiently blunt the rise in HR compared to controls (mean baseline: 225/min ± 4) leading to early discontinuation of this dose level. Four mg/kg rhDAO_mHBM (mean baseline HR rhDAO_mHBM: 233/min ± 5) significantly mitigated the increase in HR compared to buffer (*p* = 0.008; Fig. 3a). MAP decreased slightly in all groups during the 30-min histamine infusion (buffer 64 mmHg ± 3.3; 4 mg/kg DAO 61 mmHg ± 0.8). The changes in MAP appeared to differ during two short periods between the treatment groups. There was a more pronounced drop in MAP between 20 and 25 min in the group receiving buffer, and a short-lasting rebound rise in MAP over baseline in this group immediately after the histamine infusion was stopped (Fig. 3b).

The body temperature decreased continuously by > 1 °C in the control group after 60 min (mean baseline 36.7 °C ± 0.34 to 35.3 °C ± 0.2), an effect that was significantly mitigated (*p* = 0.02) by the 4 mg/kg rhDAO_mHBM dose (mean baseline 37.6 °C ± 0.09 to 37.1 °C ± 0.05) (Supplementary Fig. 2a).

After intravenous infusion of 2 and 4 mg/kg rhDAO_mHBM initial peak plasma concentrations were 20 and 33 µg/mL after 10 min, and 13 and 23 µg/mL respectively for the remainder of the experiment (Fig. 3c).

A continuous infusion of 8 µg/kg/min histamine increased histamine plasma concentrations to 28.7 ng/mL (± 4.9) in control animals at minute 40 but only to 3.1 ng/mL (± 0.2) and 1.9 ng/mL (± 0.3) in the 2 mg/kg and 4 mg/kg rhDAO_mHBM groups, respectively (Fig. 3d). Plasma histamine concentrations were significantly lower in the guinea pigs receiving 4 mg/kg rhDAO_mHBM compared to controls (*p* = 0.002).

Baseline median urinary histamine and 1-Methylhistamine concentrations were 118 ng/mL (range 17–854 ng/mL) and 190 ng/mL (range: 43–1022 ng/mL) respectively. When those histamine concentrations were normalized to creatinine concentrations (range 4–76 mg/dL) in the urine spot samples, the concentration ranges narrowed down to 1–11 ng/mL for histamine and 2–22 ng/mL for 1-Methylhistamine per mg/dL creatinine (Fig. 3c, d). Histamine infusions of 8 µg/kg/min (equivalent to a cumulative dose of 240 µg/kg over 30 min) increased the median urinary histamine concentrations to 388 ng/mL (range 220–1360 ng/mL; paired *t*-test: *p* = 0.02), and the median 1-Methylhistamine concentrations to 444 ng/mL (range 359–2080 ng/mL; paired *t*-test: *p* = 0.006). When normalized for urinary excretion of creatinine this was an 8.6-fold (range 6.6–14.9) and 4.0-fold (range 3.9–6.5) increase in their concentrations respectively.

The excess histamine excretion caused by continuous infusion of 8 µg/kg/min histamine was reduced by 2 mg/kg rhDAO_mHBM and was abrogated by 4 mg/kg rhDAO_mHBM to a 1.3-fold and 0.7-fold increase in histamine and 1-Methylhistamine respectively (Fig. 3e, f). The rise of urinary histamine and its metabolite were highly significantly different between the control and rhDAO_mHBM treatment for histamine/creatinine (*p* = 0.004) and 1-Methylhistamine/creatinine (*p* < 0.0001).

Under oxygenation, no apparent differences were observed in the partial concentration of O_2_ in the arterial blood (Supplementary Fig. 3a). Partial concentrations of CO_2_ (pCO_2_) increased from minute 0–60 in all study groups (Supplementary Fig. 3c). This rise in pCO_2_ was expected considering the pulmonary complications often associated with narcosis in guinea pigs. Finally, histamine infusion did not lower tissue oxygenation, because lactate concentrations remained similar between groups (Supplementary Fig. 3b).

## Discussion

Histamine is probably the most important mediator causing acute pathological effects in human anaphylaxis with plasma concentrations rising > 100-fold in severe allergic reactions to, for example, contrast media or hymenoptera stings [11, 13]. Low to moderately severe allergic reactions with modest levels of histamine release can be treated by H1 receptor blockers [11]. However, studies with histamine infusion in men showed that already a 3 to sixfold increase in histamine levels may override the limited protective effects of H1 or H2 receptor blockers or a combination of both [22, 23]. Concentrations of, e.g., diphenhydramine, often used as an intravenous H1R blocker, reaches steady-state concentrations of 0.4 µM in skin and plasma, which includes 90% of the drug being protein bound [24]. Local histamine concentrations reach 10 to 1000 µM strongly indicating that the effects of histamine can unlikely be prevented by antihistamines in patients with severe reactions [11, 25, 26].

In humans, a histamine infusion rate of 0.25 µg/kg/min increases plasma histamine levels to > 2 ng/mL, which already induces a 30–40% increase in HR [22]. Our experiments demonstrate that ~ tenfold higher infusion rates (> 2 µg/kg) are required to produce sustained tachycardia in guinea pigs (Fig. 2).

The present experiments in guinea pigs show that sustained tachycardia is induced after histamine levels rise by about 5–10 ng/mL. While histamine levels rise further during continued histamine infusion, the apparent maximal HR seemed to reach a plateau. This was due to a technical property of the used device, which only measured a maximum heart rate of 300 beats per minute. Histamine also induces tachycardia in humans. Intravenous infusions of histamine linearly increased the HR by approximately 15–95% when plasma histamine levels increased from 1 ng/mL (upper normal limit) to 5 ng/mL [22].

The key finding in this study was that pretreatment with 4 mg/kg rhDAO_mHBM protected guinea pigs from histamine-induced hemodynamic effects at histamine infusion rates of 8 µg/kg/min. This infusion rate increased histamine plasma concentrations in untreated guinea pigs to 40 ng/mL (or more in the pilot experiments). Such plasma concentrations of histamine are typically encountered only during severe allergic reactions, e.g., after hymenoptera stings in humans, which induced an average fall in MAP of 50 mmHg (i.e., shock) or during mast cell activation events in mastocytosis patients [13, 27].

As the histamine-degrading capacity of the enzyme rhDAO_mHBM is not species-dependent, rhDAO_mHBM may be able to treat severe cardiovascular responses during anaphylaxis in humans. The effects on blood pressure were limited in guinea pigs despite high histamine concentrations, confirming species differences in the effects of histamine on cardiovascular parameters [23]. For example, histamine does not increase the HR in anesthetized dogs despite its blood pressure-lowering effects [23]. However, a previous study in guinea pigs showed a dose-dependent decrease in MAP by about 10 mmHg after bolus infusions of 8 µg/kg histamine together with a small drop in HR [28]. It is likely that counter-regulation by the sympathetic nervous system is responsible for the different reaction patterns under continuous histamine infusion [29]. This sympathetic counter-regulation may be also responsible for the transient rebound in MAP after cessation of histamine infusion in control animals, which did not occur when rhDAO_mHBM was used.

In recent experiments, our group used the same intravenous dose of 4 mg/kg rhDAO_mHBM in mice after receiving 5 mg/kg histamine subcutaneously. rhDAO_mHBM reduced the maximum plasma histamine levels from ~ 800 to 200 ng/ml, reduced the induced drop in body temperature from  – 4.4 °C to  – 1.6 °C, and mitigated clinical symptoms [15]. The present data show a significantly lower body temperature (*p* = 0.02) at the end of the histamine infusion  in guinea pigs receiving buffer compared to 4 mg/kg rhDAO_mHBM (Supplementary Fig. 2a). These data support the concept that the histamine-degrading capacity of rhDAO_mHBM is species independent. These studies corroborate that rhDAO_mHBM ameliorates histamine-induced pathophysiological signs and symptoms even at very high histamine concentrations.

Recombinant human wild-type DAO has a dominant short alpha half-life of 3.7 min in rats. The alpha half-life can be almost completely eliminated by mutation of the HBM increasing the area under the curve 33-fold [14]. The infusion of 2 mg/kg of rhDAO_mHBM in guinea pigs resulted in DAO concentrations of approximately 8–12 µg/mL for at least 100 min. Doubling the dose of rhDAO_mHBM to 4 mg/kg increased its levels to 23–33 µg/mL. This abrogated the increase in plasma histamine concentrations and thereby prevented the hemodynamic changes caused by the continuous infusion of 8 µg/kg/min histamine (Fig. 3d).

In contrast, the 2 mg/kg dose of rhDAO_mHBM did not prevent the histamine-induced increase in HR. We therefore abandoned further testing of this dose after two animals. The data obtained indicate a threshold for the clinical benefit of DAO between 2 and 4 mg/kg in the current animal experiments. This threshold effect is supported by urinary excretion of histamine which was prevented by the 4 mg/kg dose but incompletely by the 2 mg/kg dose of rhDAO_mHBM.

Urinary concentrations of histamine and 1-Methylhistamine, the histamine metabolite of histamine-N-methyltransferase (HNMT) increased several-fold after the end of the histamine challenge at 60 min. When pretreating guinea pigs with 4 mg/kg rhDAO_mHBM before the histamine challenge, 1-Methylhistamine levels were significantly lower, indicating a shift in histamine catabolism from HNMT to DAO. When calculating the total excretion of histamine and 1-Methylhistamine in urine after 60 min, less than 2% of the infused histamine was excreted by the kidneys. The disappearance of > 95% of histamine was also observed in mice, which showed much lower histamine plasma concentrations than expected [15]. In previous mouse experiments, a similar rapid shift of intravenously infused radioactive histamine from the blood to other compartments was published [30, 31]*.* Only 5% of radioactive histamine was found in the blood compartment after 2.5 min.

As histamine plasma levels normalized ten minutes after histamine infusion had been discontinued, we assumed that the half-life of histamine in guinea pigs is in the range of minutes and is comparable to the two minutes reported in humans [32]. A histamine half-life of 3.7 min is derived from this study assuming first-order elimination using the formula: [remaining quantity after time t] = [initial quantity] * ((1/2) ^ ([time] / [half-life])) and the mean histamine values of our control group (minute 40 with 28.1 ng/mL and minute 50 with 4.5 ng/mL). This value is comparable to published human data [32, 33].

The higher tolerability of guinea pigs to infused histamine compared to humans might be mediated by the large amounts of DAO in the liver. DAO concentrations in the guinea pig liver are higher than in any other organ and the enzyme is easily accessible to circulating blood [34]. In guinea pigs, humans, and rats intravenous infusion of heparin leads to a nearly complete release of DAO from its stores into circulation within minutes [35–37]. In this study, baseline levels of circulating DAO in guinea pigs were at the limit of detection, while heparin increased the circulating endogenous DAO activity in guinea pigs > 24-fold, which is comparable to pregnant women [38]. Therefore, the endogenous levels of DAO even during pregnancy are insufficient to protect against high systemic histamine concentrations, consistent with reports that severe anaphylaxis can occur during pregnancy or peripartum [39].

The present study was able to identify an appropriate dose of DAO to prevent histamine-induced hemodynamic effects in guinea pigs. These observed effects might be translated to humans and will in the future help guide dosing rhDAO_mHBM to demonstrate its efficacy in humans during histamine challenge studies.

### Supplementary Information

Below is the link to the electronic supplementary material.

Supplementary file1 (PDF 692 KB)

## Data Availability

The data that support the findings of this study are available from the corresponding author, BJ, upon reasonable request.
