# Supplementary material for: Recombinant human diamine oxidase prevents hemodynamic effects of continuous histamine infusion in guinea pigs
Source: Inflamm Res. 2023 Oct 9;72(10-11):2013–22. doi: 10.1007/s00011-023-01783-3 (PMC10611646; doi:10.1007/s00011-023-01783-3)
Supplement: Supplementary file 1 — Supplementary file1 (PDF 692 KB) [file 11_2023_1783_MOESM1_ESM.pdf]

Supplement to

Recombinant human diamine oxidase prevents haemodynamic effects of continuous histamine infusion in guinea pigs

Matthias **Weiss-Tessbach**[1,2], Birgit **Reiter**[3], Elisabeth **Gludovacz**[4], Thomas **Boehm**[1], Bernd **Jilma**[1*], Marlene **Rager-Resch**[1]

[1] Department of Clinical Pharmacology, Medical University Vienna, Vienna, Austria

[2] Department of Medicine I, Division of Infectious Diseases and Tropical Medicine, Medical University Vienna, Vienna, Austria

[3] Department of Laboratory Medicine, Medical University Vienna, Vienna, Austria

[4] Department of Biotechnology, University of Natural Resources and Life Sciences, Vienna, Austria

Published in: Inflammation Research

*Corresponding author

Bernd Jilma, MD

Department of Clinical Pharmacology, Medical University Vienna

Waehringer Guertel 18-20, 1090 Vienna, Austria

Tel.: +43 40400 29810

Email: bernd.jilma@meduniwien.ac.at

Orcid: 0000-0001-5652-7977

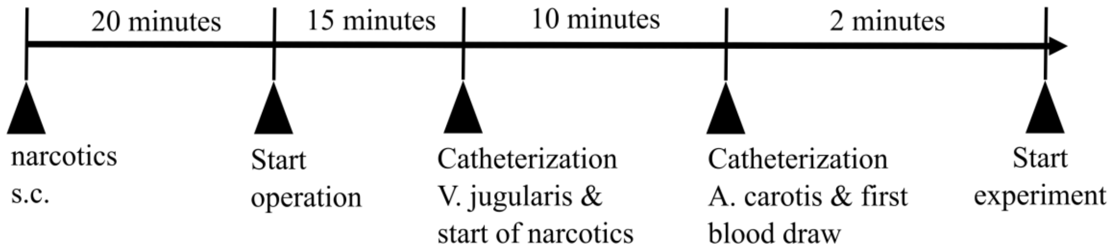

Supplementary Figure 1: Pre-experimental procedure.

Abbreviations: s.c. = subcutaneous, V. = vena, A. = arteria

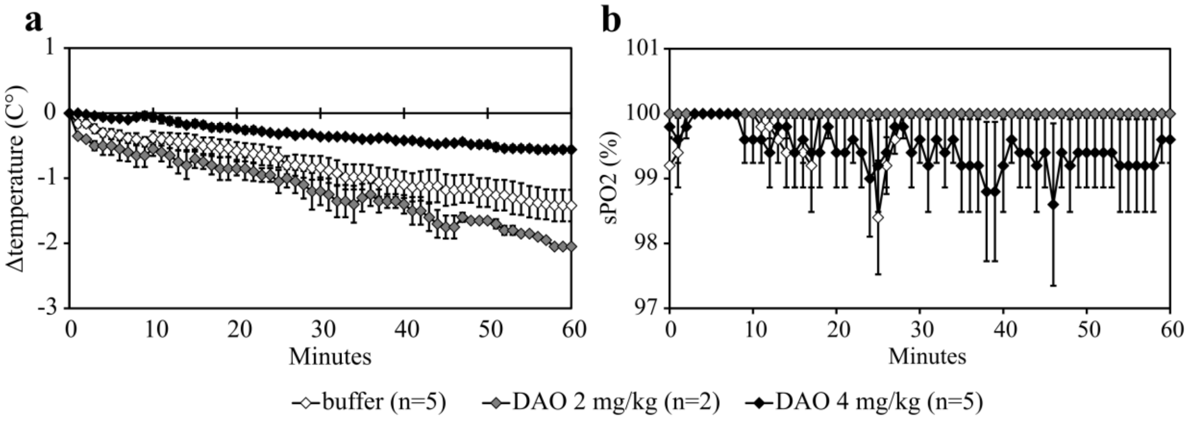

Supplementary Figure 2: Body temperature reduction and peripheral oxygen saturation

In guinea pigs treated with 8 µg/kg/min histamine body temperature did not differ between buffer and 2 mg/kg rhDAO_mHBM and 2 mg/kg and 4 mg/kg pretreated animals, but significantly differed between buffer and 4 mg/kg ($p=0.02$). Rectal temperature and peripheral oxygen saturation measurements are presented as mean ±SEM. Area under the curves of temperature changes compared to baseline were calculated and differences between groups were compared using an unpaired t-test.

Lactate and arterial blood gases during continuous histamine infusion in guinea pigs.

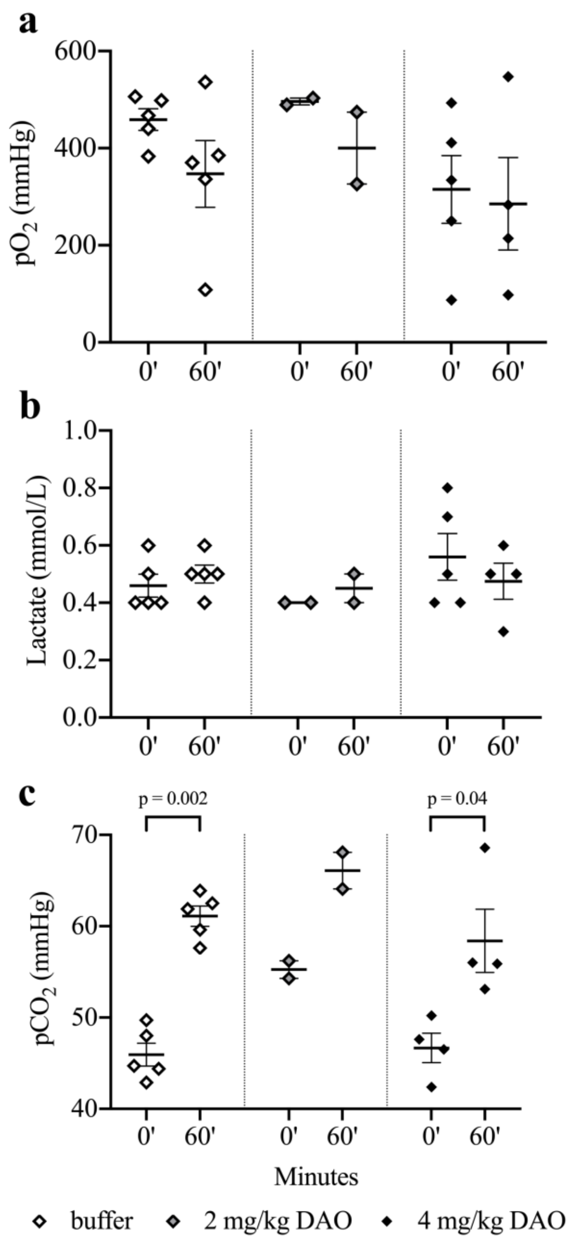

Supplementary Figure 3: Lactate and arterial blood gases at baseline and 60 minutes after the start of the experiment. Guinea pigs received 8 µg/kg/min histamine with either buffer (n=5), 2 mg/kg rhDAO_mHBM (n=2) or 4 mg/kg (n=5) rhDAO_mHBM. Individual measurements are presented as diamonds with their respective mean ± SEM. P-values were calculated using a paired t-test.

Histamine and 1-Methylhistamine measurements using liquid chromatography – tandem mass spectrometry (LC-MS/MS) in plasma and urine

Histamine base and d4-histamine dihydrochloride were obtained from Sigma-Aldrich (San Francisco, CA, USA) and 1-Methylhistamine hydrochloride from Cayman Chemicals (Ann Arbor, MI, USA).

All samples were analyzed with a 6500 plus QTrap system equipped with a TurboIon Source for electrospray ionization. The chromatographic system consisted of an Exion LC AD (Sciex, Framingham, MA, USA) equipped with a Cortecs UPLC HILIC 1.6 µm, 2,1 mm x 100 mm column (Waters, Milford, MA, USA).

Gradient elution was performed using 100 mM ammonium formate adjusted to pH 3 with formic acid as mobile phase A and acetonitrile as mobile phase B at a flow rate of 0.55 mL/min and an oven temperature of 45°C. Quantification was performed using multiple reaction monitoring (MRM) in positive ionization mode with the following mass transitions m/z 111.9 → 95.0 for histamine, 125.9 → 109.2 for N-tau histamine and m/z 115.8 → 99.0 for d4-histamine (used as an internal standard for both analytes). Urine (10 µL) was mixed with internal standard (10 µL, d4-histamine 0.3 µg/mL in water), precipitated with 900 µL acetonitrile, vortexed for 5 seconds and centrifuged at 20,800g for 5 minutes at 4°C.

Plasma (10 µL) was mixed with sample (10 µL) and with 110 µL acetonitrile with d4-histamine (2.5 ng/mL), vortexed for 5 sec and centrifuged at 20,800g for 5 min at 4°C. The supernatants (5 µL) were injected into the LC-MS system. Due to the presence of all analytes in urine and plasma, respectively, calibrators and quality controls were prepared in water and 5% bovine serum albumin in PBS buffer respectively.

For urine samples calibration ranges of 10 to 250 ng/mL for histamine and 50 to 1000 ng/mL for 1-Methylhistamine and for plasma samples a range of 2.5 to 150 ng/mL were used.

Plasma histamine concentrations below the limit of quantification were classified as half limit of quantification.
